# The Influence of Specialist Palliative Care in Aggressive End of Life Management of Patients with Advanced Cancer

**DOI:** 10.2478/sjph-2026-0007

**Published:** 2026-03-01

**Authors:** Nena Golob, Teja Oblak, Boštjan Šeruga

**Affiliations:** University of Ljubljana, Faculty of Medicine, Vrazov trg 2, 1000 Ljubljana, Slovenia; Institute of Oncology Ljubljana, Department of Acute Palliative Care, Zaloška cesta 2, 1000 Ljubljana, Slovenia; Institute of Oncology Ljubljana, Epidemiology and Cancer Registry, Zaloška cesta 2, 1000 Ljubljana, Slovenia; Institute of Oncology Ljubljana, Division of Medical Oncology, Zaloška cesta 2, 1000 Ljubljana, Slovenia

**Keywords:** Anticancer treatment, Medical care, Specialist palliative care, End of life, specifično onkološko zdravljenje, ostala zdravstvena oskrba, specializirana paliativna oskrba, zadnje obdobje življenja

## Abstract

**Introduction:**

There is a growing concern that terminally ill cancer patients may be receiving aggressive management at the end of life. This study aimed to evaluate the use of aggressive management (anticancer treatment and medical care) in patients with advanced cancer in their last month of life and to evaluate the influence of specialist palliative care on it.

**Methods:**

This retrospective study included adult patients with advanced solid cancers treated at the Institute of Oncology Ljubljana who died between January 2015 and December 2019. Multiple logistic regression models were used to assess the association between the aggressiveness of anticancer treatment and medical care, the year of death, age at death, sex, prognosis, type of cancer and inclusion of specialist palliative care.

**Results:**

We included 1,736 patients in our analysis. 538 (31%) patients received at least one anticancer treatment modality. There was an increasing use of chemotherapy and novel systemic therapies. A significant predictor for aggressive anticancer treatment (OR 0.96; 95% CI 0.95–0.97) and medical care (OR 0.96; 95% CI 0.95–0.97) was younger age. Inclusion into the specialist palliative care was strongly associated with less aggressive anticancer treatment (OR 0.19; 95% CI 0.12–0.31) and medical care (OR 0.25; 95% CI 0.15–0.40).

**Conclusions:**

In the last month of life, there was an increasing use of chemotherapy and novel systemic therapies, especially in younger patients. Inclusion in specialist palliative care was associated with less aggressive end-of-life management.

## Izvlečekk

**Uvod:**

Ob napredku onkologije in razmahu terapevtskih možnosti je zaskrbljenost, da so bolniki z napredovalim rakom pogosto deležni agresivne obravnave v zadnjem obdobju življenja, upravičena. Namen raziskave je bil oceniti uporabo agresivne obravnave (specifičnega onkološkega zdravljenja in ostale zdravstvene oskrbe) pri bolnikih z napredovalim (razsejanim/razširjenim) rakom v zadnjem mesecu življenja ter oceniti vpliv specializirane paliativne oskrbe nanjo.

**Metode:**

V retrospektivno raziskavo so bili vključeni odrasli bolniki z napredovalimi solidnimi raki, ki so se zdravili na Onkološkem inštitutu Ljubljana in umrli med januarjem 2015 in decembrom 2019. Z multivariatno logistično regresijo smo ugotavljali povezanost med agresivnim specifičnim onkološkim zdravljenjem in ostalo agresivno zdravstveno oskrbo v zadnjem mesecu življenja in posameznimi pojasnjevalnimi spremenljivkami (letom smrti, spolom, starostjo ob smrti, vrsto raka, prognozo raka in vključenostjo v specializirano paliativno oskrbo).

**Rezultati:**

V raziskavo smo vključili 1736 bolnikov. Od tega je 538 (31 %) bolnikov prejelo vsaj eno vrsto agresivnega specifičnega onkološkega zdravljenja. Uporaba kemoterapije in novih sistemskih zdravljenj se je sčasoma povečala. Mlajši bolniki so bili pogosteje deležni agresivnega specifičnega onkološkega zdravljenja (OR 0,96; 95 % IZ 0,95–0,97) in ostale agresivne zdravstvene oskrbe (OR 0,96; 95 % IZ 0,95–0,97) kot starejši bolniki. Vključitev v specializirano paliativno oskrbo je pomembno prispevala k manj agresivni obravnavi – prejetju agresivnega specifičnega onkološkega zdravljenja (OR 0,19; 95 % IZ 0,12–0,31) in ostale agresivne zdravstvene oskrbe (OR 0,25; 95 % IZ 0,15–0,40) – bolnikov z napredovalim rakom v zadnjem mesecu življenja.

**Zaključki:**

V zadnjem mesecu življenja se je sčasoma povečala uporaba kemoterapije in novih sistemskih zdravljenj, zlasti pri mlajših bolnikih z napredovalim rakom. Vključitev v specializirano paliativno oskrbo je bila močno povezana z manj agresivno obravnavo bolnikov ob koncu življenja.

## 1 INTRODUCTION

For patients with advanced cancer, anticancer treatment may be considered if it enhances the quality of life, regardless of its effect on patients’ survival (1, 2). Within palliative care (PC), timely discontinuation of aggressive anticancer therapy is essential to avoid unnecessary harm (3, 4). Consistent with international guidelines, early integration of PC alongside standard oncology treatment improves the quality of life and reduces aggressive interventions near the end of life (EoL) (1,2,3,4,5). Overtreatment may be detrimental, leading to adverse effects, delayed EoL discussions, and compromised quality of life (5).

The recent expansion of systemic anticancer therapies has raised concerns about increasingly aggressive EoL care (6, 7). Aggressive interventions often conflict with patient preferences, complicate caregiver bereavement, and provide limited healthcare value (8, 9).

To assess the aggressiveness of EoL care, several indicators have been proposed, including: administration of anticancer treatment at the EoL; deaths and hospitalisations at the EoL in acute care facilities/intensive care units (ICUs); emergency room visits at the EoL; and late hospice referral (10, 11). The provision of specific oncological treatment at the EoL is often influenced by factors related to the patient, family, disease, and treating physician characteristics (12, 13). PC involvement improves the EoL care, reducing hospitalisations, intensive care use, and hospital deaths (3, 4).

Few studies have evaluated the EoL use of novel systemic therapies (STs) – targeted therapy (TT), immunotherapy (IT) – as well as radiotherapy (RT) and surgery. Our study aimed to assess the aggressiveness of anticancer treatment and medical care in patients with advanced solid cancers in the last month of life in the main Slovenian oncology centre and to explore possible predictors for the aggressive management, with a focus on the influence of the specialist PC services that are gradually growing throughout the country.

## 2 METHODS

### 2.1 Data sources and patient cohort

**Figure 1.**
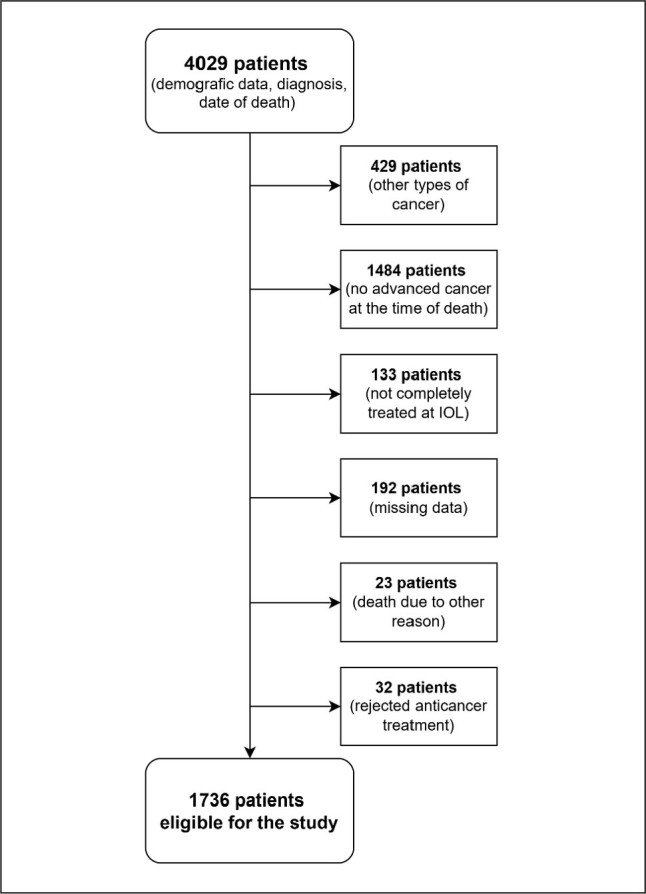
Flow chart of the assessment of patients for eligibility. IOL – Institute of Oncology Ljubljana.

This retrospective study evaluated the aggressiveness of anticancer treatment and medical care in adult patients with advanced solid cancers (head and neck cancer, germ cell carcinoma and sarcoma (other); gastrointestinal; lung; breast; gynaecologic; or genitourinary cancer treated at the Institute of Oncology Ljubljana (IOL), who died between January 1, 2015 and December 31, 2019. Demographic and diagnostic data for patients residing in the broader Ljubljana area, with a population of approximately 330,000, were obtained from the Slovenian Cancer Registry. The electronic health records (EHRs) of these patients were reviewed to determine study eligibility (Figure 1).

The study was approved by the National Medical Ethics Committee of the Republic of Slovenia on January 7, 2021 (0120-484/2020/4).

### 2.2 Outcome measures and statistical analysis

Any anticancer treatment administered in the last month of life – ST (chemotherapy (ChT), IT, TT and other biological therapy (defined as a form of systemic treatment that uses agents derived from living organisms to target molecular or immune-mediated mechanisms selectively), RT or surgery – was considered a measure of aggressive anticancer treatment. Aggressive medical care was defined as admission to the ICU in the last month of life, death in the ICU, and/or in-hospital death.

Data were independently reviewed for accuracy by two investigators; however, formal inter-rater reliability statistics were not calculated, as discrepancies were resolved through consensus.

Descriptive analyses were performed to summarise demographic and clinical characteristics. A multiple logistic regression model was used to evaluate the association between aggressive anticancer treatment (ST, RT, or surgery) in the last month of life and possible predictors. Age was modelled as a continuous variable, and odds ratios (ORs) are reported per one-year increase.

A similar analysis assessed the association between aggressive medical care and the same covariates. In addition, the potential influence of a specialist PC approach on both measures of aggressiveness was examined. Cancer prognosis was categorised as good, intermediate and poor according to the 5-year net survival for solid cancers in Slovenia during 2012–2016 (14).

Sensitivity analyses using alternative definitions of aggressive anticancer treatment and medical care were conducted to assess the robustness of our results. To evaluate potential clustering by cancer type, we additionally performed a sensitivity analysis restricted to patients with lung cancer, the largest subgroup in our analytical cohort.

All statistical analyses were performed using IBM® SPSS® Statistics, version 29.0. ORs with corresponding 95% confidence intervals (CIs) were reported. A P-value < 0.05 was considered statistically significant. No formal correction for multiple comparisons was applied; therefore, the overall Type I error rate across tests may be inflated, and statistically significant findings should be interpreted with caution.

## 3 RESULTS

### 3.1 Eligible patient cohort

The initial search identified 4,029 potentially eligible patients. After reviewing the EHRs 2,293 patients were excluded for the following reasons: 429 patients were diagnosed with cancer, not included in the study; 1,484 patients had an early-stage disease; 133 patients received treatment at various institutions; 192 patients had incomplete EHRs; 23 patients died of causes unrelated to cancer, and 32 patients declined anticancer treatment.

### 3.2 Patients’ characteristics

A total of 1,736 patients were included in the analysis, of whom 868 (50.0%) were women. The median age at death was 70 years (IQR 62–78), with ages ranging from 18 to 98 years.

Regarding cancer type, 542 patients (31.2%) died from lung, 320 (18.4%) from gastrointestinal, 288 (16.6%) from genitourinary, 274 (15.8%) from breast, 204 (11.8%) from other and 108 (6.2%) from gynaecologic cancers.

Based on 5 year net survival, prognosis at the time of death was categorised as good in 572 patients (32.9%), intermediate in 424 (24.4%), and poor in 740 (42.6%).

### 3.3 Aggressive anticancer treatment and medical care

Overall, 538 patients (31.0%) received at least one modality of anticancer treatment – ST, RT or surgery – in the last month of life. Of all treatments administered, 273 (48.0%) were ST, 270 (47.5%) RT, and 26 (4.6%) surgery. The proportion of patients receiving any anticancer treatment increased from 27.6% (109/395) in 2015 to 32.3% (101/313) in 2019.

**Figure 2.**
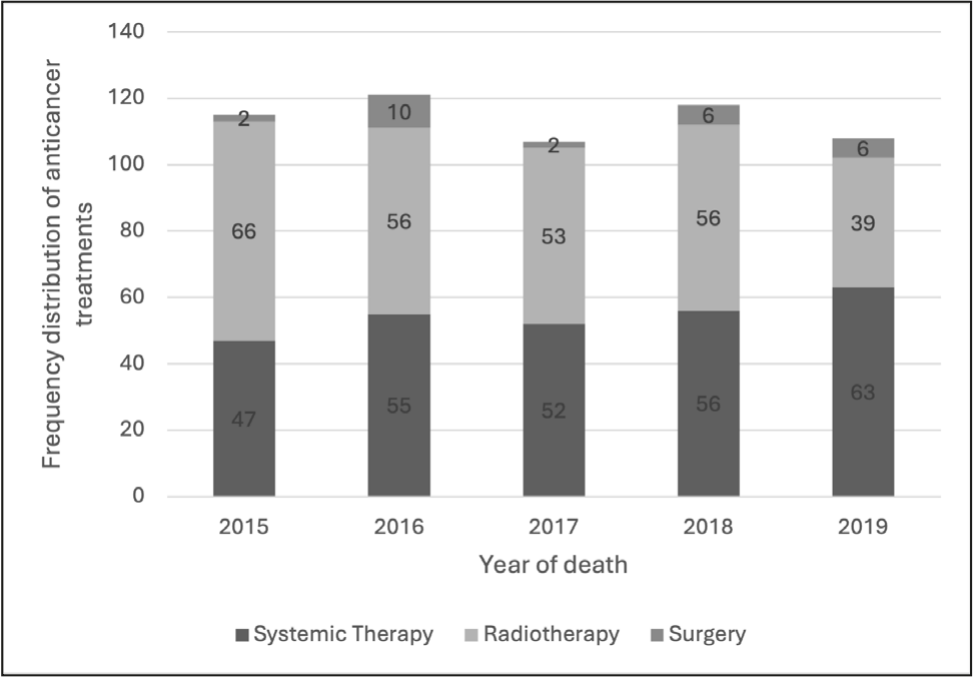
Frequency distribution of different anticancer treatments in the last month of life. * Patients receiving different modalities of treatment are included in all treatment groups corresponding to the therapies they received. 31 patients received a combination of modalities of treatment in the last month of life: 4 received systemic therapy and surgery, 26 received systemic therapy and radiotherapy, and one (1) patient received surgery and radiotherapy.

The proportion of patients receiving RT decreased from 16.7% (66/395) in 2015 to 12.5% (39/313) in 2019, while surgery was rare, used in 0.5% (2/395) of patients in 2015 and in 1.9% (6/313) in 2019 (Figure 2).

A total of 273 patients received ST in the last month of life, and 24 received two different types of ST. The most frequently prescribed ST was ChT, administered to 182 patients (10.5%). ChT use increased from 9.6% (38/395) in 2015 to 12.1% (38/313) in 2019 (Figure 2). The proportion of patients receiving novel ST also rose, from 3.2% (13/395) in 2015 to 5.4% (29/313) in 2019.

During the study period, 620 (35.7%) patients experienced at least one form of aggressive medical care: 28 (1.6%) were admitted to the ICU, 19 (1.1%) were admitted and died in the ICU, and 586 (33.7%) died in acute hospital care. Thirteen patients experienced more than one modality of aggressive medical care. The proportion of patients receiving aggressive medical care varied across years, from 33.2% (131/395) in 2015 to 35.1% (110/313) in 2019.

In-hospital specialist PC was provided to 237 patients (13.7%). Annual proportions ranged from 14.7% (58/395) in 2015 to 13.7% (43/313) in 2019.

### 3.4 Predictors of aggressive anticancer treatment and medical care

Older patients had significantly lower odds of receiving aggressive anticancer treatment (OR 0.96; 95% CI 0.95–0.97) and medical care (OR 0.96; 95% CI 0.95–0.97) in the last month of life as compared with younger patients (Tables 1 and 2). The OR of 0.96 per one-year increase in age corresponds to the OR of 0.66 per 10-year increase in age. Patients who received aggressive anticancer treatment were also more likely to undergo aggressive medical care (OR 1.84; 95% CI 1.47–2.30) (Table 2). Inclusion into the specialist hospital PC was associated with substantially lower odds of receiving aggressive anticancer treatment (OR 0.19; 95% CI 0.12–0.31) and medical care (OR 0.25; 95% CI 0.15–0.40) in the last month of life as compared with standard oncologic care (Tables 1 and 2). Sex, year of death, cancer type (results for gynaecological cancers are inconclusive) and prognosis were not significantly associated with the receipt of anticancer treatment and medical care in the last month of life (Tables 1 and 2).

**Table 1.** Multiple logistic regression analysis exploring the association between different predictors and the use of aggressive anticancer treatment in the last month of life.

	**P**	**OR**	**95% CI**
**Gender (male = REF)**	0.53	0.92	0.71 – 1.19
Age at death^*^	< 0.001	**0.96**	**0.95 – 0.97**
**Year of death**	0.12	1.06	0.99 – 1.15
**Prognosis of cancer**			
Low (REF)	/	/	/
Intermediate	0.22	1.28	0.86 – 1.92
High	0.80	1.07	0.65 – 1.75
**Type of cancer**			
Others (REF)	/	/	/
Gastrointestinal	0.38	0.82	0.53 – 1.28
Lung	0.20	1.34	0.86 – 2.07
Breast	0.35	1.28	0.76 – 2.15
Gynecological	0.05	**0.53**	**0.28 – 1.00**
Urogenital	0.87	0.87	0.55 – 1.39
**Specialist palliative care (No = REF)**	< 0.001	**0.19**	**0.12 – 0.31**

Legend:

* Age was included as a continuous variable; odds ratios (OR) correspond to a one-year increase in age.

P – P-value, OR – odds ratio, CI – confidence interval, REF – reference.

**Table 2.** Multiple logistic regression analysis exploring the association between different predictors and aggressive medical care in the last month of life.

	**P**	**OR**	**95% CI**
**Gender (male = REF)**	0.61	0.93	0.72 – 1.21
Age at death*	< 0.001	**0.96**	**0.95 – 0.97**
**Year of death**	0.12	1.06	0.98 – 1.15
**Prognosis of cancer**			
Low (REF)	/	/	/
Intermediate	0.30	1.24	0.83 – 1.85
High	0.85	1.05	0.64 – 1.73
**Type of cancer**			
Others (REF)	/	/	/
Gastrointestinal	0.41	0.83	0.53 – 1.30
Lung	0.20	1.34	0.86 – 2.08
Breast	0.43	1.24	0.73 – 2.09
Gynecological	0.04	**0.52**	**0.28 – 0.97**
Urogenital	0.55	0.87	0.55 – 1.38
**Aggressive oncological treatment (Yes vs. No)**	< 0.001	**1.84**	**1.47 – 2.30**
**Specialist palliative care (No = REF)**	< 0.001	**0.25**	**0.15 – 0.40**

Legend:

* Age was included as a continuous variable; odds ratios (OR) correspond to a one-year increase in age.

P – P-value, OR – odds ratio, CI – confidence interval, REF – reference.

Sensitivity analyses did not substantially change the results. When aggressive anticancer treatment was defined as ST or RT alone, older age and inclusion into the specialist PC remained significantly associated with lower odds of receiving aggressive anticancer treatment (age: OR 0.98; 95% CI 0.97–0.99 for both definitions; specialist PC: OR 0.12; 95% CI 0.06–0.25 and OR 0.41; 95% CI 0.24–0.69, respectively). Similarly, when aggressive medical care was defined as death in an acute hospital setting, which accounted for 93% of all aggressive medical care events, older age (OR 0.97; 95% CI 0.96–0.98), inclusion into the specialist PC (OR 0.04; 95% CI 0.02–0.10), and absence of aggressive anticancer treatment (OR 1.57; 95% CI 1.26–1.95) remained significantly associated with lower odds of aggressive medical care. In addition, in analysis restricted to patients with lung cancer, older age (OR 0.96; 95% CI 0.94–0.98) and inclusion into the specialist PC (OR 0.12; 95% CI 0.04–0.34) were associated with significantly lower odds of receiving aggressive anticancer treatment.

## 4 DISCUSSION

The therapeutic landscape for patients with advanced cancer has expanded considerably in recent years, raising concerns regarding the aggressiveness of anticancer treatment and medical care at the EoL (6, 15). Our study is the first study in Eastern Europe providing a comprehensive evaluation of the aggressiveness of management of patients with advanced cancer in the last month of life. In the last month of life, there was an increasing use of ChT and novel STs, especially in younger patients. Inclusion into the specialist PC resulted in substantially less aggressive management at the EoL.

### 4.1 Aggressive anticancer treatment

We observed an increasing use of ST at the EoL, including both ChT and novel agents such as TT and IT (Figure 2).

ChT remains a cornerstone of treatment for patients with advanced cancer. In our cohort, 10.5% of patients received ChT in the last month of life, with an increasing trend over time (Figure 2). This proportion is lower than previously reported rates of 11.7–37%, indicating that treatment practices at our institution may more closely reflect current best-practice recommendations (3, 12, 16,17,18). This finding is consistent with our other study assessing the aggressiveness of care in the last two weeks of life (13).

The use of novel ST – particularly oral TT and IT – is rising and persists later in the disease trajectory, even within the EoL setting (19, 20). In our study, TT exceeded IT use and demonstrated a consistent upward trend. The availability of oral agents and their perception as less aggressive treatment than intravenous ChT may encourage oncologists to inappropriately continue this therapy closer to death (21, 22).

The observed trends in the use of ST underscore the need for institutional policies and evidence-based guidelines that balance active therapy with quality of life considerations in terminally ill patients (1, 5).

### 4.2 Aggressive medical care

Reported ICU admission rates at the EoL vary substantially, ranging from 2% to 30%, with fewer than 10% in only a minority of studies (7, 16, 23). In our cohort, ICU admission was notably rare (1.6%), a rate consistent within hospice populations (24).

ICU mortality at the EoL has been less frequently documented. Only two studies have reported these outcomes, with markedly divergent results (47.7% vs. 5%) (11, 23). In our cohort, ICU deaths were very uncommon (1.1%), underscoring the limited use of aggressive interventions at the EoL at the IOL.

Published data report high (40% to 65%) hospital death rates (25, 26). In contrast, in our study, only 35.8% of patients died in acute hospital care and only 1% of those receiving specialist PC.

The lower proportion of hospital deaths observed in our cohort compared with international reports may reflect differences in study populations, health-system organisation, and timing of treatment de-escalation.

### 4.3 Predictors to receive aggressive management

#### 4.3.1 Patients’ age

In our cohort, younger age was significantly associated with aggressive anticancer treatment and medical care at the EoL (Tables 1 and 2), consistent with prior studies reporting reduced ChT use in older patients (3, 12, 15, 27, 28).

This association likely reflects greater treatment tolerance, clinician willingness to continue disease-directed therapy, and patient or family preferences in younger individuals, whereas comorbidities and functional decline in older patients may prompt earlier treatment de-escalation (3, 5, 9, 12, 15, 27, 28).

No association was observed between aggressive management at the EoL and cancer prognosis (Tables 1 and 2). Although prognosis is central to treatment decision-making, it is often difficult to estimate it accurately in routine clinical practice, particularly across heterogeneous tumour types (3, 27).

To our knowledge, no prior studies have systematically evaluated the prognosis of cancer as a potential predictor. This factor is relevant given the heterogeneous prognosis of cancer types within the same organ system and the tendency of oncologists to manage specific organ system where individual practice style can influence treatment decisions (12, 33, 29, 30).

#### 4.3.2 Specialist palliative care

In our cohort, 237 of 1,736 patients (13.7%) were included in the hospital-based specialist PC. PC involvement was associated with less aggressive anticancer treatment and medical care, consistent with prior evidence that PC reduces intensive EoL interventions (Tables 1 and 2) (31, 32). Overall, our findings emphasise the importance of integration of PC to mitigate the risk of aggressive anticancer treatment and medical care interventions at the EoL (1, 2, 4).

PC is a holistic approach that enhances the quality of life of patients and their families facing progressive and incurable disease by addressing physical, psychological, social, and spiritual challenges (33). Early PC initiation reduces the use of ChT near the EoL, lengthens the interval between the last ChT and death, lowers the rate of invasive interventions, improves quality of life, may prolong survival, and decreases healthcare costs (3, 34, 35).

Cessation of anticancer treatment at the EoL is complex, influenced by patient/family hope for disease control, physician discomfort with stopping treatment, prognostic uncertainty, and psychosocial factors (5, 12, 36, 37). Oncologists often overestimate the expected survival of patients with advanced cancer (38, 39). In some countries, financial incentives encourage the continuation of ST, whereas in Slovenia, anticancer treatments and PC services are fully publicly funded, with no additional reimbursement for oncologists for prescribing ST (12, 16, 40).

According to the National Palliative Care Program, PC is regionally organised in Slovenia at the primary – generalist and specialist levels (41, 42). In Slovenia, there are now 14 specialist PC services in eight statistical regions (Central Slovenia, Upper Carniola, Drava, Savinja, Mura, Coastal-Karst, Gorizia, and Southeast Slovenia). Currently, they are mainly offering mobile PC residential visits and phone support for patients in late PC, where, in case of a cancer diagnosis, specific anticancer treatment has already been discontinued.

However, at the time of the study, access to the specialist PC services was very limited. They started to develop after 2021. Currently, there are two hospital-based specialist PC services in Slovenia that also provide early PC.

Our findings show that enrolling patients with advanced cancer into the specialist PC at the IOL protects them from aggressive anticancer treatment and medical care at EoL. Thus, we urge incentives for specialist PC services in Slovenia to broaden their activities and to provide, besides residential visits in the late disease stage, also other types of support – department services, outpatient clinics, hospital consultative services – for patients with various health conditions and complex and difficult-to-manage symptoms and specific needs still receiving disease-oriented treatment. These services are already accessible to patients with advanced cancer at the IOL.

To our knowledge, this is the largest study to date in our region assessing the management of patients with cancer in the last month of life. Previously, we reported results of an analysis of the last two weeks of life in the same population of patients, which similarly support the integration of PC to limit aggressive management at the EoL (13).

However, our study has several limitations. First, its retrospective design relies on the accuracy of data entered into EHRs and does not capture clinicians’ judgment or decision-making processes. Second, aggressive anticancer treatment and aggressive medical care were defined as composite variables. It may be argued that certain interventions, such as single-fraction RT at the EoL or death in acute hospital care, do not necessarily represent aggressive management. We therefore conducted several sensitivity analyses using alternative definitions of aggressive management at the EoL, none of which substantially changed our results. In addition, to assess potential clustering within cancer types, we performed a sensitivity analysis restricted to patients with lung cancer, which likewise yielded stable findings. Furthermore, information on the primary indication for RT, fractionation, and treatment intent (palliative vs. disease-directed) was incomplete and inconsistently documented in our dataset, precluding reliable identification and reclassification of patients receiving single-fraction symptom-relief RT.

Third, several potentially important confounders (e.g., time since diagnosis, performance status, symptom burden, comorbidities, and social factors) could not be included because of missing or unreliable data. Other relevant indicators of aggressive medical care, such as ER visits and hospice referrals, were also not studied. Although ER visits were initially planned for inclusion, legal and administrative barriers prevented access to these data. Moreover, Slovenia currently has only one operating hospice, located in Ljubljana, which primarily provides PC during the very final phase of life (usually within the last month before death); therefore, hospice referrals were not included in the analysis.

Fourth, although the use of novel STs is increasing, only 91 patients in our cohort received these treatments, limiting conclusions regarding contemporary trends. Similarly, the modest increase in aggressive care observed between 2015 and 2019 involved small absolute numbers. The proportion of patients receiving specialist PC decreased slightly during the study period due to a temporary staff shortage, which represented an additional limitation. Importantly, specialist PC during the study period was predominantly initiated late in the disease course, typically after discontinuation of anticancer treatment. As a result, the potential benefits of earlier integration of specialist PC during active disease management could not be fully assessed. It is plausible that earlier involvement of specialist PC would have resulted in an even greater reduction in aggressive anticancer treatment and medical care at the EoL.

Finally, our findings may not be fully generalisable to other settings or healthcare systems. Nevertheless, in Slovenia, the IOL is the country’s leading national oncology centre, providing care to approximately 70% of all cancer patients, making it the primary institution for oncology care nationwide.

## 5 CONCLUSIONS

This retrospective single-institution study examined aggressive anticancer treatment and medical care in the last month of life in patients with advanced solid cancers treated at the OIL, the country’s main cancer centre. 31% of patients received anticancer treatment. The use of ChT and newer ST (TT, IT) has increased over time, especially in younger patients. Patients who received aggressive cancer treatment were also at higher risk of receiving aggressive medical care. Inclusion into the specialist PC resulted in less aggressive cancer treatment and medical care. Authors urge both clinicians and policymakers to adopt an early PC approach in patients with advanced cancer and to invest in the development of the PC network in Slovenia.
